# Beta-blockers for the prevention of sudden cardiac death in heart failure patients: a meta-analysis of randomized controlled trials

**DOI:** 10.1186/1471-2261-13-52

**Published:** 2013-07-13

**Authors:** Muaamar Al-Gobari, Chadia El Khatib, François Pillon, François Gueyffier

**Affiliations:** 1 Laboratoire de Biologie et Biométrie Evolutive - Equipe Modélisation des Effets Thérapeutiques, UMR 5558 Université Claude Bernard Lyon1, Rue Guillaume Paradin, Bp8071, 69376 Lyon Cedex 08, France; 2 Service de Pharmacologie Clinique et essais thérapeutiques, Hospices Civils de Lyon, Lyon Cedex 08, France

**Keywords:** Beta, Blocker, Sudden cardiac death, Heart failure, Meta, Analysis

## Abstract

**Background:**

In many studies, beta-blockers have been shown to decrease sudden cardiac death (SCD) in heart failure patients; other studies reported mixed results. Recently, several large randomized control trials of beta blockers have been carried out. It became necessary to conduct a systematic review to provide an up-to-date synthesis of available data.

**Methods:**

We conducted a meta-analysis of all randomized controlled trials examining the use of beta-blockers vs. placebo/control for the prevention of SCD in heart failure patients. We identified 30 trials, which randomized 24,779 patients to beta-blocker or placebo/control. Preferred Reporting Items for Systematic Reviews and Meta-Analyses (PRISMA) guidelines were followed. Eligible studies had to be randomized controlled trials and provide information on the incidence of sudden cardiac death in heart failure patients. Additional inclusion criteria included: treatment for >30 days and follow-up ≥ 3 months. Studies of patients <18 years, randomization to beta-blocker vs. an angiotensin converting enzyme (without placebo) and/or beta-blocker in both arms were excluded from the analysis. Pre-specified outcomes of interest included SCD, cardiovascular death (CVD), and all-cause mortality and were analyzed according to intention-to-treat.

**Results:**

We found that beta-blockers are effective in the prevention of SCD [OR 0.69; 95% CI, 0.62–0.77, P < 0.00001], cardiovascular death (CVD) [OR 0.71; 95% CI, 0.64–0.79, P < 0.00001], and all-cause mortality [OR 0.67; 95% CI, 0.59–0.76, P < 0.00001]. Based on the study analysis, 43 patients must be treated with a beta-blocker to prevent one SCD, 26 patients to prevent one CVD and 21 patients to prevent all-cause mortality in one year.

**Conclusion:**

Beta-blockers reduce the risk of sudden cardiac death (SCD) by 31%, cardiovascular death (CVD) by 29% and all-cause mortality by 33%. These results confirm the mortality benefits of these drugs and they should be recommended to all patients similar to those included in the trials.

## Background

Sudden cardiac death is defined as a non-violent death that cannot be explained, occurring less than 24 hours from the onset of symptoms [1]. Sudden cardiac death accounts for 300 000 to 400 000 deaths annually in the United States, depending on the definition used [2,3]. When restricted to death <2 hours from the onset of symptoms, 12% of all natural deaths were classified as sudden in one study, and 88% of those were due to cardiac disease [2,3]. Sudden cardiac death is the most common and often the first manifestation of coronary heart disease and is responsible for ≈50% of the mortality from cardiovascular disease in the United States and other developed countries [4]. The risk of sudden cardiac death (SCD) is most pronounced among patients with heart failure, in whom the 1 year absolute risk of SCD is between 4 and 13% [5]. It is worth mentioning that BEST [6], a randomized trial of the beta-blocker bucindolol in patients with advanced chronic heart failure, reported that it did not reduce sudden cardiac death and/or all-cause mortality. However, BEST included demographically diverse groups and severe heart failure patients [7]. In this study, we intended to quantify the effect of beta-blockers in the risk reduction of sudden cardiac death in patients with heart failure by using pooled analysis techniques. Recently, several large randomized control trials of beta-blockers have been carried out. Therefore, a systematic review is required to provide an up-to-date synthesis of available data.

## Methods

### Study search

We searched the Cochrane Central Register of Controlled Trials (Central) in the Cochrane Library (Version 2012) and MEDLINE (1966 to March 2012). The bibliographies of identified studies were checked. The Medline query was limited to studies involving human subjects, randomized controlled trials and/or meta-analyses. No language restrictions were applied.

### Selection criteria and data abstraction

A systematic review of the literature with meta-analysis was needed to identify all clinical trials evaluating beta-blockers for heart failure and reporting all-cause mortality. Eligible studies had to be placebo-controlled trials and provide information on the incidence of sudden cardiac death. Additional inclusion criteria included: treatment for >30 days and follow-up ≥3 months. Studies of patients <18 years, randomization to beta-blocker vs. an angiotensin converting enzyme (without placebo), and/or beta-blockers in both arms were excluded from the analysis.

**Figure 1:**
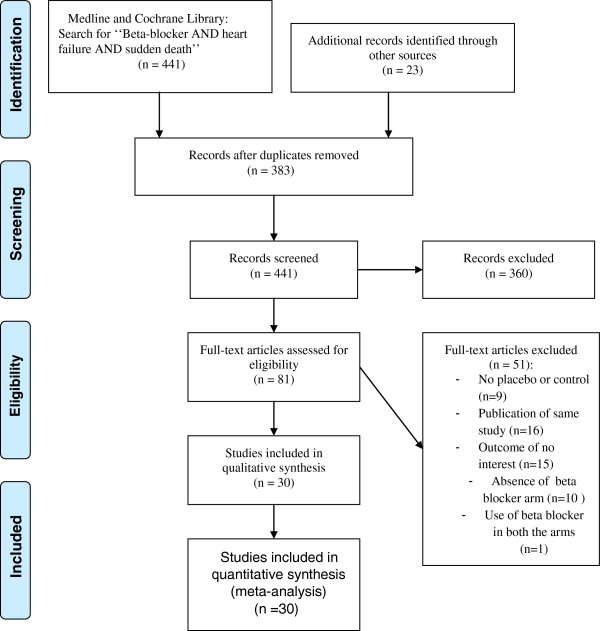
**PRISMA flow diagram for the meta-analysis.** Study selection process according to Preferred Reporting Items for Systematic Reviews and Meta-Analyses (PRISMA) guidelines.

Abstracted data included eligibility criteria, baseline characteristics, study design (including treatment and control arms), follow-up, and outcomes. Pre-specified outcomes of interest included SCD, cardiovascular death (CVD), and all-cause mortality. Outcomes were analyzed according to intention-to-treat. Study quality was formally evaluated using the Jadad score [8] for the quality assessment of randomized controlled trials. For the purpose of this analysis, studies which had a score of 3/5 or more were considered high quality. The study selection process (according to the PRISMA guidelines) is shown in Figure 1.

### Statistical analysis

The patient was chosen as the individual unit of analysis (as opposed to person years). The effects of beta-blockers on SCD, CVD, and all-cause mortality were determined using fixed-effect and random-effect modeling. Fixed-effect modeling was performed using the Mantel and Haenszel method. Random-effect modeling was conducted using the DerSimonian and Laird method [9]. The results were similar with both methods, so we only reported the random-effect results. Treatment effect was measured using odds ratios (ORs) with 95% confidence intervals (CIs).

Heterogeneity across the studies was estimated using I-square test [9]. I-square values of 25%, 50%, and 75% correspond to low, moderate, and high levels of heterogeneity [10]. Meta-analysis results were reported only if the I-square value was under 75%. Sensitivity analyses were performed for each outcome measure to assess the contribution of each study to the pooled estimate by excluding individual trials one at a time and recalculating the combined OR for the remaining studies. Statistical testing was two-tailed, and statistical significance was declared with α = 0.05. All analyses were conducted using RevMan software (Version 5.1).

## Results

### Search results

After searching Medline and the Cochrane Library, we identified 441 abstracts which were reviewed for inclusion and exclusion criteria (Figure 1). Out of these, 361 were excluded for the following reasons: non-randomized study (including observational studies, pharmacokinetic and pharmacodynamic studies, substudies, editorials, etc.; n=349), absence of placebo or inactive control arm (n=11), and inclusion of subjects < 18 years (n=1).

The full manuscripts of the remaining 81 studies were retrieved for detailed review. Following full manuscript review, an additional 51 studies were excluded: no placebo or control (n= 9), duplicate report or substudy (n= 16), absence of a beta-blocker arm (n=10), outcome of no interest (n=15) and use of beta-blockers in both arms (n= 1).

### Trial characteristics and study quality

As shown in Table 1, we identified 30 randomized controlled trials of beta-blocker for inclusion in this meta-analysis, which enrolled a total of 24,779 patients [6,11-39]. The mean follow-up duration was 11.51 months (0.96 year) and all trials are placebo controlled except the trial of Anderson et al. [11] which used standard therapy. Using the Jadad score [8], all studies were estimated with score of 3–5 and qualified as high quality. All trials were analyzed according to the intention-to treat paradigm.

**Table 1:** Randomized trials of beta blockers for the prevention of sudden cardiac death

**Trial (Reference)**	**Year**	**Number of patients**	**Name of drug**	**Comparator**	**Daily maintenance dose (mg)**	**Follow-up (months)**	**Jadad scoring**
Anderson et al.^12^	1985	50	Metoprolol	Control	61	19	3
ANZ^16^	1997	415	Carvedilol	Placebo	12.5	19	4
BEST^6^	2001	2708	Bucindolol	Placebo	152	24	5
BHAT	1986	3837	Propranolol	Placebo	180/240	25	5
Bristow et al.^17^	1994	139	Bucindolol	Placebo	12.5/50/200	3	5
Bristow et al.^18^	1996	345	Carvedilol	Placebo	12.5/25/50	6	5
Capricorn^19^	2001	1959	Carvedilol	Placebo	50	15.6	5
CIBIS II^21^	1999	2647	Bisoprolol	Placebo	10	15	5
CIBIS^20^	1994	641	Bisoprolol	Placebo	5	23	5
CILICARD	2000	124	Celiprolol	Placebo	100	12	5
Colucci et al.^22^	1996	366	Carvedilol	Placebo	100	15	5
COPERNICUS^8^	2002	2289	Carvedilol	Placebo	50	10.4	5
de Milliano et al.	2002	59	Metoprolol	Placebo	150	6	5
ELANDD	2011	116	Nebivelol	Placebo	5/10	6	5
Engleimeir et al.^23^	1985	25	Metoprolol	Placebo	92	12	4
Fisher et al.^24^	1994	50	Metoprolol	Placebo	87	6	5
Hansteen V. et al.^25^	1982	560	Propranolol	Placebo	160	12	5
Krum et al.^26^	1995	49	Carvedilol	Placebo	50	4	5
MDC^27^	1993	383	Metoprolol	Placebo	108/115	18	3
MERIT-HF^28^	1999	3991	Metoprolol	Placebo	159/170	12	5
Metra et al.^29^	1994	40	Carvedilol	Placebo	50	4	4
Olsen et al.^30^	1995	60	Carvedilol	Placebo	81	4	5
Packer et al.^31^	1996	1094	Carvedilol	Placebo	60	6	5
Pollock et al.^32^	1990	19	Bucindolol	Placebo	200	3	4
RESOLVD	2000	426	Metoprolol	Placebo	156	6	5
SENIORS^14^	2005	2128	Nebivelol	Placebo	7.7	21	5
Sturm	2000	100	Atenolol	Placebo	89	24	5
UHLIR et al.	1997	91	Nebivelol	Placebo	2.5/5	3.5	5
Wisenbaugh et al.^15^	1993	24	Nebivelol	placebo	5	3	5
Woodley et al.^13^	1991	50	Bucindolol	Placebo	175	3	5

ANZ: Australian/New Zealand Heart Failure Research Collaborative Group; BEST: *Beta*-*blocker Bucindolol* in *patients* with *advanced chronic heart failure*; CAPRICORN: Effect of carvedilol on outcome after myocardial infarction inpatients; COPRINCUS: Effect of Carvedilol on the Morbidity of Patients with Severe chronic Heart Failure; MDC: Metoprolol in Dilated Cardiomyopathy Trial study; Merit-HF: Metoprolol CR/XL Randomized Intervention Trial in Congestive Heart Failure; SENIORS: Randomized trial to determine the effect of nebivolol on mortality and cardiovascular hospital admission in elderly patients with heart failure.

### Baseline patient characteristics

Baseline patient characteristics (Table 2) were remarkably similar in age and gender in all trials except for Woodley et al. [12] which included younger patients and the SENIORS [13] which included elderly patients.Therefore, the mean age ranged from 28–76 and all trials enrolled mostly men except for Wisenbaugh et al. [14] and ELANDD [35] which enrolled 50% and 65% women respectively. Copernicus [22] and RESOLVD [36] were not evaluated for cardiovascular death outcome due to missing data. Four trials were restricted to patients with non-ischaemic cardiomyopathy, three to ischaemic patients, three not reported, and the remainder enrolled patients with ischaemic and non-ischaemic cardiomyopathy. Mean left ventricular ejection fraction ranged from 16-62%.

**Table 2:** Patient characteristics in randomized trials of beta blockers for the prevention of sudden cardiac death

**Trial (Reference)**	**Mean age (Years)**	**Male (%)**	**Inclusion criteria**	**Population (Ischaemic or non-ischaemic)**	**Mean EF (%)**	**NYHA**
Anderson et al.	50	66	IDC	Non-ischaemic	29	II-IV
ANZ^13^	67	80	chronic heart failure	Ischaemic	29	II-III
BEST^6^	60	78	NYHA III-IV, EF ≤ 35%	Both	23	III-IV
BHAT	55	NR	MI, HF	NR	NR	NR
Bristow et al.^14^	55	61	IDC and ISCD	Both	24	I-IV
Bristow et al.^15^	60	76	Mild, moderate, chronic heart failure	Both	23	II-IV
CAPRICORN^16^	63	74	Acute MI, EF ≤ 40%	Ischaemic	32	NR
CELICARD	57	89.5	NYHA II- IV, LVEF<40%	Both	26	II-III
CIBIS II^18^	61	80	NYHA III or IV, EF ≤ 35%	Both	28	III-IV
CIBIS^17^	60	83	IDC, NYHA III-IV, ≤ 40%	Both	25	III-IV
Colucci et al.^19^	54	85	Mildly symptomatic heart failure	Both	23	II-III
COPERNICUS^20^	63.3	79.5	HF and EF ≤ 25%	67% ischaemic	20	NR
de Milliano et al.	65	60	HF,, LVEF<35%,	Both	25	II-III
ELANDD	66	35	HF, age>40 years, LVEF>45%	Non-ischaemic	62	II-III
Engleimeir et al.^21^	50	64	IDC	Both	17	II-III
Fisher et al^22^	63	96	HF and CAD	NR	23	II-IV
Hansteen V. et al.^23^	58	84.5	Acute MI	NR	NR	NR
Krum et al.^24^	55	78	Advanced heart failure	Both	16	II-IV
MDC^25^	49	73	DCM and EF<40%	Non-ischaemic	22	I-III
MERIT-HF^26^	64	77	NYHA II-IV,EF ≤ 40%	Both	28	II-IV
Metra et al.^27^	51	90	NYHA II-III, IDC	Non-ischaemic	20	II-III
Olsen et al.^28^	52	94	NYHA II-III, IDC/CAD	Both	20	II-IV
Packer et al.^29^	58	77	Chronic heart failure	Both	23	II-IV

*CAD* Coronary artery disease, *CHF* congestive heart failure, *DCM* dilated cardiomyopathy, *ISCD* ischemic dilated cardiomyopathy, *EF* ejection fraction, *HF* Heart failure, *IDC* ischemic dilated cardiomyopathy, *MDC* Metoprolol in Dilated Cardiomyopathy, *MI* myocardial infarction, *NR* not reported, *NYHA* 'Classification of' New York Heart Association.

### Efficacy of beta-blockers

**Figure 2:**
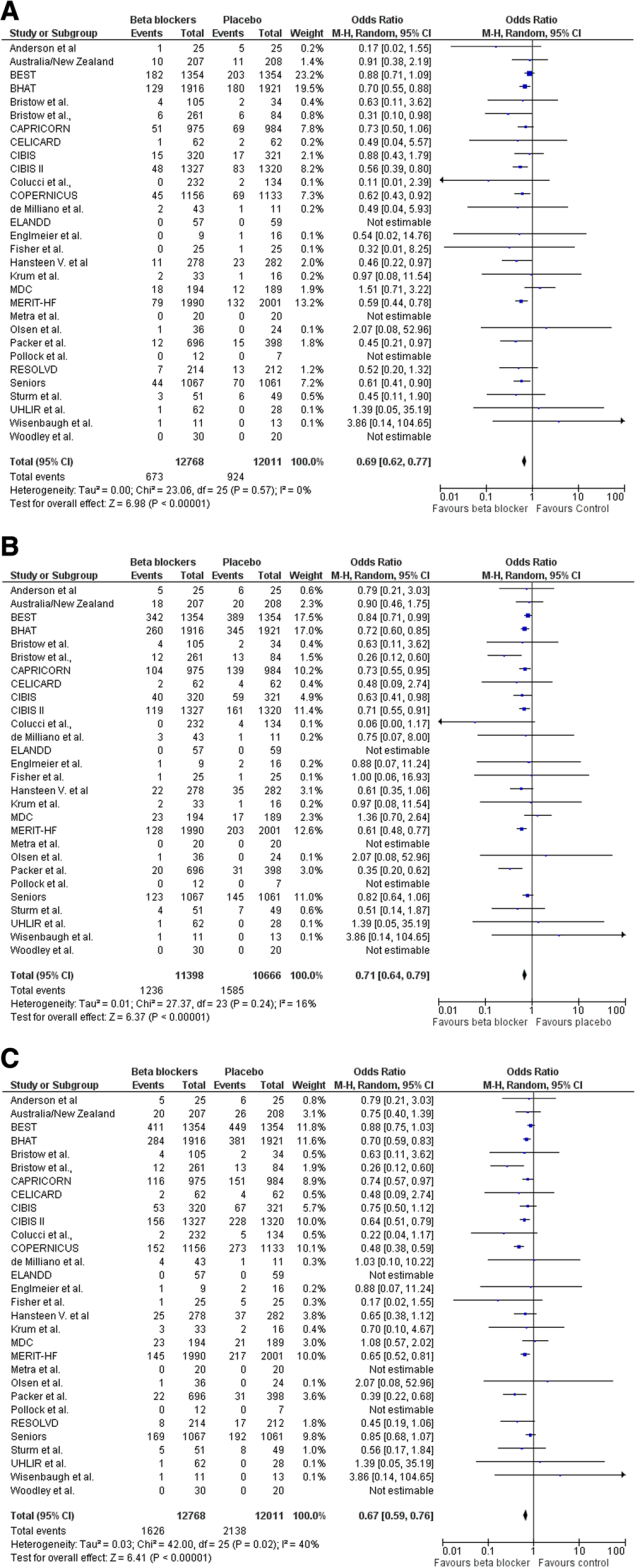
**Efficacy of beta blockers compared with control for the (A) Prevention of sudden death. (B)** Cardiovascular death, and **(C)** all-cause mortality in patients with heart failure.

A total of 3,764 deaths occurred in the 24,779 patients included in this analysis, including 1,597 SCDs. The SCD rate was 5.27% (n= 673/12768) in those treated with beta-blockers compared with 7.69% (n = 924/12011) in those treated with placebo/control [OR 0.69; 95% CI, 0.62–0.77, P < 0.00001] as shown in Figure 2(A). Cardiovascular mortality rate was 10.84% (n = 1236 /11398) in those treated with beta-blockers and 14.86% (n =1585/10666) in those assigned to placebo/control [OR 0.71; 95% CI, 0.64–0.79, P < 0.00001] see Figure 2(B). All-cause mortality rate was 12.82% (n = 1626 /12678) in those treated with beta-blockers and 17.80% (n =2138/12011) in those assigned to placebo/control [OR 0.67; 95% CI, 0.59–0.76, P < 0.00001] as shown in Figure 2(C).

**Figure 3:**
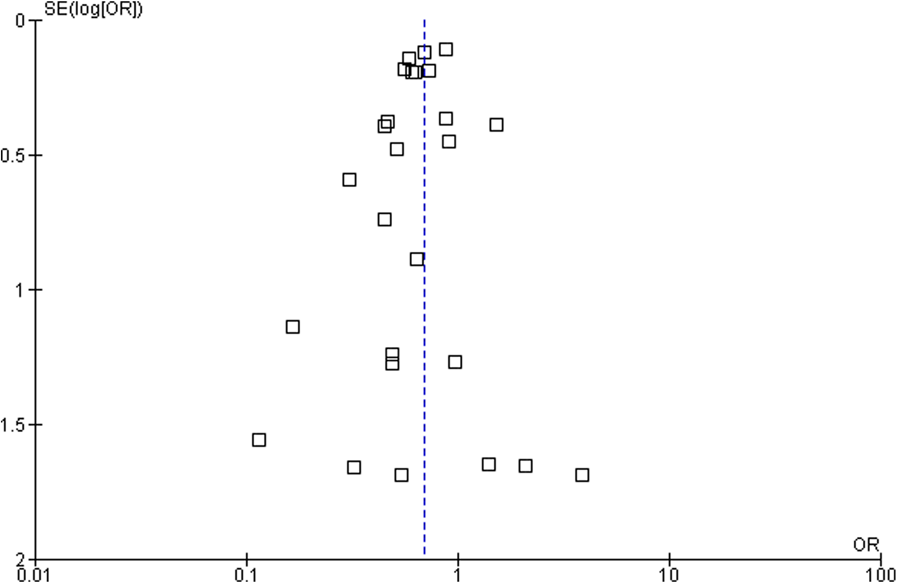
Funnel plot of SE (log odds ratio) by odds ratio to evaluate publication bias for effect of treatment in sudden cardiac death (SCD).

**Figure 4:**
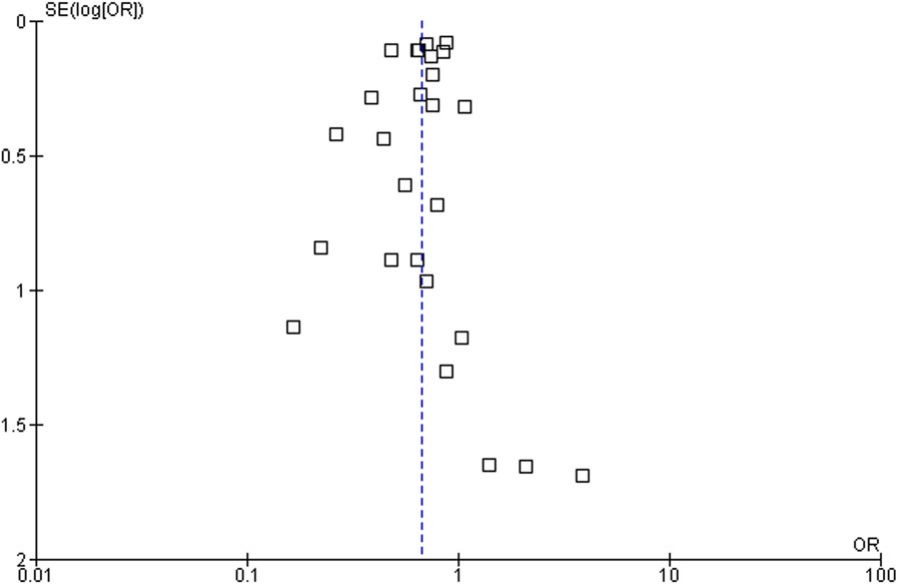
Funnel plot of SE (log odds ratio) by odds ratio to evaluate publication bias for effect of treatment for all-cause mortality.

Based on these data, 43 patients need to be treated (NNT) with beta-blockers to prevent one SCD, 26 patients to prevent one CVD, and 21 patients to prevent all-cause mortality in one year. The forest plot comparison of beta-blockers vs. placebo for SCD and all-cause mortality is shown in Figure 3 and Figure 4 respectively. The I-square test of heterogeneity was relatively low in SCD, CVD, and all-cause mortality with I^2^ =0%, 20%, and 43% respectively.

### Sensitivity analysis

The BEST trial had the largest relative overall weight of 23.2%, 17.5%, and 11.8% in SCD, CVD and all-cause mortality respectively. Therefore, we conducted a sensitivity analysis to assess the impact of this trial on the results. When excluding the BEST trial from the random-effect estimates, there was no significant difference: OR for SCD [0.64 (95% CI 0.57 -0 .72), p = 0.00001], OR for CVD [0.69 (95% CI 0.62 -0 .77), p = 0.00001] and OR for all-cause mortality [0.65 (95% CI 0.58 -0 .73), p = 0.00001]. I^2^ = 0%, 7%, and 25% respectively. The Capricorn [18] and Hansteen et al. [25] and BHAT [39] studies included patients with acute myocardial infarction. But when they were excluded from the analysis, no significant difference was found: OR for SCD [0.69 (95% CI 0.61 -0 .78), p = 0.00001], OR for CVD [0.70 (95% CI 0.60 -0 .82), p = 0.00001] and OR for all-cause mortality [0.65 (95% CI 0.55 -0 .77), p = 0.00001]. I^2^ = 0%, 26%, and 47% respectively. Also, the trial of ELANDD [35] had included patients with LVEF>45%. The sensitivity analysis showed no significant difference: OR for SCD [0.69 (95% CI 0.62 - 0 .77), p = 0.00001], OR for CVD [0.71 (95% CI 0.67–0.79), p = 0.00001] and OR for all-cause mortality [0.67 (95% CI 0.59 - 0 .76), p = 0.00001]. I^2^ = 0%, 20%, and 43% respectively.

### Publication bias

To assess a potential existence of publication bias in the effect of beta-blockers in sudden cardiac death and all-cause mortality, a funnel plot as shown in Figure 3 and Figure 4 indicates large symmetry and therefore a publication bias is likely excluded.

## Discussion

There exist several meta-analyses which evaluated the mortality benefits of beta-blockers among chronic heart failure patients [40-44]. One of the eldest is the study of Heidenreich et al. [40] that reported significant reduction in all-cause mortality but had not concluded for sudden cardiac death. This is apparently due to lack of power and sudden death missing data in the studied clinical trials. Also, the meta-analysis of McAllister et al. [41] showed 24% risk reduction of mortality related to the magnitude of heart rate reduction but not to dosing of beta-blockers. A recent meta-analysis by Chatterjee et al. [42] included 21 trials using beta-blockers in patients with heart failure and reduced ejection fraction showing a 31% reduction in overall mortality with no difference among the different agents used. However, this study, like many others, had not evaluated the overall reduction of beta-blockers in the prevention of sudden cardiac death. Another study, Fauchier et al. [44], found similar beneficial effects of beta-blockers in ischemic and non-ischemic cardiomyopathy. Though, the number of clinical trials that classified such patients accounts for <22% in our meta-analysis. Similarly, study of Bonet et al. [43] reported no difference in mortality benefits among ischemic and non-ischemic heart disease and proposed greater benefit of vasodilating beta-blockers compared with the non-vasodilating agents particularly in patients with non-ischemic cardiomyopathy and attributed mortality benefits to significant reduction of pump failure and sudden death. Briefly, previous studies whether had not evaluated overall reduction of beta-blockers in the prevention of sudden cardiac death or need to be updated such as the studies of Bonet et al. [43] and Heidenreich et al. [40] as several recent and large randomized clinical trials have been carried out.

In this meta-analysis of 24,779 randomized patients, we found that beta-blockers are effective in the prevention of SCD with [OR 0.69; 95% CI, 0.62–0.77, P < 0.00001], CVD [OR 0.71; 95% CI, 0.64–0.79, P < 0.00001], and all-cause mortality [OR 0.67; 95% CI, 0.59–0.76, P < 0.00001]. Ventricular arrhythmias (including non-sustained ventricular tachycardia) have been documented in up to 85% of patients with severe congestive heart failure [45]. As antiarrhythmic agents, beta-blockers have been shown to reduce morbidity and mortality in patients with chronic heart failure in randomized controlled trials, and consistently reduce the risk of SCD by 40–55% [20,28]. However, our meta-analysis showed a 32% reduction of SCD risk. As indicated earlier, the 1-year absolute risk of SCD in heart failure patients is 4-13% [5]. In our study, the 1-year absolute risk of SCD in the beta-blocker group and placebo/control group is 5.5% and 8.10% respectively. Mortality rates increase the higher the New York Heart Association (NYHA) class, but the proportion of patients dying suddenly (rather than from progressive pump failure) is highest among those with less severe heart failure (NYHA class II or III) [28]. The evaluation of clinical benefits for patients at different stages of heart failure by subgroup analysis merits further investigation. Our study included two clinical trials with acute myocardial infarction patients. When they were excluded from the meta-analysis, no significant differences were found in a sensitivity analysis of the remaining trials. Our study provides a high level of evidence given the large number of randomized patients included.

### Clinical implications

American College of Cardiology (ACC), American Heart Association (AHA), and European Society of Cardiology (ESC) guidelines recommend the use of beta-blockers to reduce sudden death and especially in patients with heart failure [46,47]. Our results support such recommendations with a high level of argument.

## Conclusion

Out of all antiarrhythmic agents, only beta-blockers have been shown to be effective at reducing the risk of SCD. Beta-blockers reduce the risk of SCD by 31%, CVD by 29%, and all-cause mortality by 33% and therefore, this meta-analysis study confirms beta-blockers’ clinical benefits and should be recommended to all patients similar to those included in the trials.

## Abbreviations

ANZ: Australia/New Zealand Heart Failure Study; BEST: Beta-blocker evaluation survival trial; CAPRICORN: Carvedilol post-infarct survival control in LV dysfunction study; CIBIS I: Cardiac insufficiency bisoprolol study; CIBIS-II: Cardiac insufficiency bisoprolol study II; COPERNICUS: Carvedilol prospective randomized cumulative survival study; HF: Heart failure; LV: Left ventricular; MDC: Metoprolol in idiopathic dilated cardiomyopathy study; MDC: Metoprolol in dilated cardiomyopathy trial study; MERIT-HF: Metoprolol CR/XL randomized intervention trial in congestive heart failure; MI: Myocardial infarction; NYHA: New York Heart Association; RCTs: Randomized controlled trials; SENIORS: Study of the effects of nebivolol intervention on outcomes and rehospitalisation in seniors with heart failure study.

## Competing interests

No conflict of interest is declared.

## Authors’ contributions

MA and FG participated in the conception and design of the study. MA, FP and CK extracted the data. MA drafted the study. MA, FP and FG had critically analyzed and interpreted the data. All authors read and approved the final manuscript.

## Pre-publication history

The pre-publication history for this paper can be accessed here:

http://www.biomedcentral.com/1471-2261/13/52/prepub
